# Causal effects of antibody-mediated immune responses on allergic diseases: A bidirectional Mendelian randomization study

**DOI:** 10.1097/MD.0000000000045796

**Published:** 2025-11-14

**Authors:** Lina Leng, Tao Xu, Quanyi Tang, Ying Li, Xiaoli Li

**Affiliations:** a Department of Rheumatology, Xingtai People’s Hospital, Xingtai, Hebei Province, China; b Department of Internal Medicine, Graduate School of Hebei North University, Zhangjiakou, Hebei Province, China; c Department of Internal Medicine, Graduate School of Hebei Medical University, Shijiazhuang, Hebei Province, China; d Department of Oncology, 82 Group Hospital of Chinese People’s Liberation Army, Baoding, Hebei Province, China.

**Keywords:** allergic diseases, allergic urticaria, antibody-mediated immune response, asthma, atopic dermatitis, Mendelian randomization

## Abstract

Accumulating evidence indicates that microbial infections and their elicited immune responses may contribute to allergic disease pathogenesis, but the causal relationships remain unclear. We performed a bidirectional 2-sample Mendelian randomization (MR) study utilizing genome-wide association study summary statistics. We analyzed causal relationships between 46 antibody-mediated immune responses and 5 allergic diseases: allergic asthma (AA), allergic conjunctivitis, atopic dermatitis (AD), allergic rhinitis (AR), and allergic urticaria (AU). The primary analysis method was inverse-variance weighted, supplemented by MR-Egger, weighted median, and weighted mode methods. Robustness was assessed through tests for heterogeneity, pleiotropy, and leave-one-out sensitivity analysis. As an observational genetic methodology, MR estimates are subject to potential biases from pleiotropy. Thus, our findings suggest associations rather than definitive causation. Polyomavirus 2 JC VP1 antibody levels and Antihuman herpes virus 7 IgG seropositivity may influence the risk of AA disease. *Toxoplasma gondii* p22 antibody levels may confer protection against allergic conjunctivitis. Epstein–Barr virus EBNA-1 antibody levels are positively associated with AD, while *Chlamydia trachomatis* momp A antibody levels are negatively associated with AD. *H pylori* CagA antibody levels are positively linked to AR. Epstein–Barr virus EBNA-1 antibody levels are inversely related to AR. The positivity of *H pylori* Catalase antibody levels and herpes simplex virus 2 mgG-1 antibody levels are potential risk factors for AU. Antihuman herpes virus 6 IE1B IgG seropositivity may be a protective factor for AU. Reverse MR indicated that AA may elevate anti-*H pylori* IgG seropositivity. Sensitivity analyses confirmed robustness (*P* > .05). Our findings unveil potential causal links between antibody-mediated immune responses and allergic diseases, which may inform future mechanistic research and therapeutic strategies, highlighting the role of pathogen-immune interactions.

## 1. Introduction

Allergic diseases refer to a group of illnesses caused by abnormal immune responses to typically harmless substances such as pollen, food, etc. These diseases mainly include allergic (or atopic) asthma (AA), allergic conjunctivitis (AC), atopic dermatitis (also known as eczema; AD), allergic rhinitis (also known as hay fever; AR), and allergic urticaria (AU).^[1–3]^ In recent years, the incidence of allergic diseases worldwide has sharply increased, especially in children and adolescents, with approximately 81 million cases of asthma and 5.6 million cases of AD diagnosed in children.^[4]^ According to the World Health Organization, asthma alone caused approximately 4,55,000 deaths worldwide in 2019,^[5]^ posing a significant challenge to public health.

The immune pathogenesis of allergic diseases is multifactorial and complex.^[6–10]^ Recent evidence suggests that microbial infections and their immune responses significantly contribute to the pathogenesis of these allergic diseases. For example, pathogen infections such as Epstein–Barr virus and polyomavirus can induce the host to produce specific antibodies, thereby affecting the balance of the immune system and, in some cases, causing allergic diseases.^[11,12]^ *H pylori* infection may have a certain degree of protective effect on AA and AD.^[13,14]^ However, recent studies show no significant correlation between *H pylori* infection and AA.^[15]^ The above contradictory clinical research results further complicate the impact of immune response induced by microbial infection on the incidence rate and prognosis of allergic diseases, and this relationship needs further research.

Antibody-mediated immune response (AIR) encompasses immune responses involving antibodies, which play a crucial role in defending against pathogens. Evaluating the response of antibodies to infection is a common method for studying the relationship between AIR and diseases. Seropositivity can indicate prior infection and help researchers understand the pathophysiological links between infections and noncommunicable diseases.^[16]^ However, traditional observational studies are often influenced by confounding factors such as environment, lifestyle, and dietary habits, which can seriously hinder the ability to determine causal relationships between risk factors and outcomes. Mendelian randomization (MR) provides a reliable method for inferring causal relationships. MR uses genetic variation as an instrumental variable (IV) to estimate the impact of exposure on outcomes.^[17]^ This method can reduce confounding factors such as social and economic environment, effectively reduce bias in causal estimation, and strengthen causal reasoning.^[18,19]^

In this study, we utilized publicly available genome-wide association study (GWAS) summary data and employed bidirectional 2-sample MR to evaluate causal relationships between AIR and allergic diseases. Thus, providing new insights and scientific evidence for the diagnosis, prevention, and personalized treatment of these diseases, and offering valuable references for clinical practice.

## 2. Materials and methods

### 2.1. Research design

**Figure 1.**
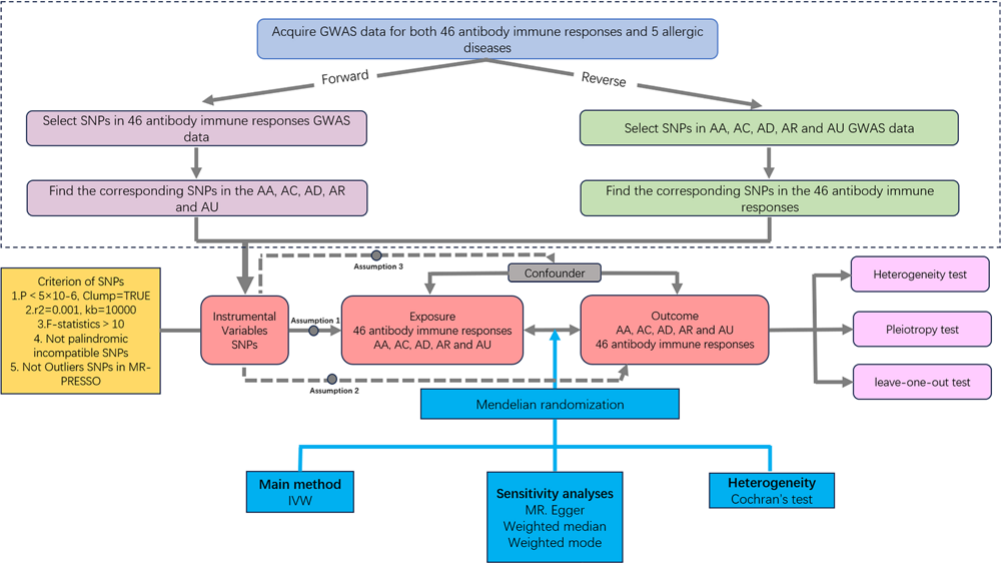
Schematic overview of the Mendelian randomization (MR) study design and its core assumptions. The workflow of the bidirectional 2-sample MR analysis conducted in this study, from the acquisition of genome-wide association study (GWAS) summary data to the causal inference and sensitivity analyses. AA = allergic asthma, AC = allergic conjunctivitis, AD = atopic dermatitis, AR = allergic rhinitis, AU = allergic urticaria, IV = instrumental variable, IVW = inverse-variance weighted, GWAS = genome-wide association study, MR = Mendelian randomization, MR-PRESSO = Mendelian randomization-the pleiotropic residual sum and outlier test, SNP = single-nucleotide polymorphism.

In MR analysis, single-nucleotide polymorphisms (SNPs) are used as IVs. These IVs need to satisfy the 3 basic assumptions described in Figure 1: association: a strong correlation between the SNP and the exposure; independence: no correlation between the SNP and any confounders that might affect the exposure‐outcome relationship; and exclusion: the SNP affects the outcome exclusively through the exposure.^[20]^ MR analysis strictly follows the STROBE guidelines to ensure the rigor and reproducibility of the results.^[21]^ We extracted consolidated information from openly accessible databases (UK Biobank, FinnGen), which had received informed consent from the participants and had been granted ethical approval.

### 2.2. Data source

Summary data related to AIR from the GWAS catalog (https://www.ebi.ac.uk/gwas/). Guillaume Butler-Laporte et al conducted serological measurements on 9724 adults of European descent using the UK Biobank, selecting 13 infectious pathogens from 46 GWAS (GWAS IDs: GCST90006884 to GCST90006909).^[16]^ It identified multiple loci associated with these immune traits, including genome-wide important loci within the major histocompatibility complex on chromosome 6 and 60 other loci outside of major histocompatibility complex.

The summary statistical data of allergic diseases comes from FinnGen GWAS (https://r12.finngen.fi/). The number of cases and controls for each phenotype is as follows: AA (13,450 cases, 2,70,290 controls), AC (29,791 cases, 4,70,557 controls), AD (31,245 cases, 4,32,874 controls), AR (15,569 cases, 4,74,650 controls), and AU (3315 cases, 4,82,901 controls). Refer to Tables 1 and 2 for details. This study selected exposure and outcomes from 2 different individuals in the UK and Finland to minimize sample overlap and ensure robustness of the results. The genetic background of the participants in this study is limited to European-ancestry, including both male and female individuals. The original study of the selected GWAS dataset was approved by the ethics committee, and all participants in the original study obtained informed consent. The dataset used in this study is publicly accessible, therefore it is deemed unnecessary to obtain ethical approval.

**Table 1:** Characteristics of the genome-wide association study (GWAS) datasets for antibody-mediated immune responses (exposures).

	GWAS ID	Trait name
Exposure	GCST90006909	Human herpes virus 7 U14 antibody levels
Exposure	GCST90006910	Anti-*Helicobacter pylori* IgG seropositivity
Exposure	GCST90006911	*Helicobacter pylori* CagA antibody levels
Exposure	GCST90006912	*Helicobacter pylori* Catalase antibody levels
Exposure	GCST90006913	*Helicobacter pylori* GroEL antibody levels
Exposure	GCST90006914	*Helicobacter pylori* OMP antibody levels
Exposure	GCST90006915	*Helicobacter pylori* UREA antibody levels
Exposure	GCST90006916	*Helicobacter pylori* VacA antibody levels
Exposure	GCST90006917	Anti-herpes simplex virus 1 IgG seropositivity
Exposure	GCST90006918	Herpes simplex virus 1 mgG-1 antibody levels
Exposure	GCST90006919	Anti-herpes simplex virus 2 IgG seropositivity
Exposure	GCST90006920	Herpes simplex virus 2 mgG-1 antibody levels
Exposure	GCST90006921	Anti-polyomavirus 2 IgG seropositivity
Exposure	GCST90006922	Polyomavirus 2 JC VP1 antibody levels
Exposure	GCST90006923	Anti-Merkel cell polyomavirus IgG seropositivity
Exposure	GCST90006924	Merkel cell polyomavirus VP1 antibody levels
Exposure	GCST90006925	Anti-*Toxoplasma gondii* IgG seropositivity
Exposure	GCST90006926	*Toxoplasma gondii* p22 antibody levels
Exposure	GCST90006927	*Toxoplasma gondii* sag1 antibody levels
Exposure	GCST90006928	Anti-varicella zoster virus IgG seropositivity
Exposure	GCST90006929	Varicella zoster virus glycoproteins E and I antibody levels
Exposure	GCST90006885	BK polyomavirus VP1 antibody levels
Exposure	GCST90006886	Anti-*Chlamydia trachomatis* IgG seropositivity
Exposure	GCST90006887	*Chlamydia trachomatis* momp A antibody levels
Exposure	GCST90006888	*Chlamydia trachomatis* momp D antibody levels
Exposure	GCST90006889	*Chlamydia trachomatis* pGP3 antibody levels
Exposure	GCST90006890	*Chlamydia trachomatis* PorB antibody levels
Exposure	GCST90006891	*Chlamydia trachomatis* tarp-D F1 antibody levels
Exposure	GCST90006892	*Chlamydia trachomatis* tarp-D F2 antibody levels
Exposure	GCST90006893	Anti-cytomegalovirus IgG seropositivity
Exposure	GCST90006894	Cytomegalovirus pp28 antibody levels
Exposure	GCST90006895	Cytomegalovirus pp52 antibody levels
Exposure	GCST90006896	Cytomegalovirus pp150 antibody levels
Exposure	GCST90006897	Anti-Epstein–Barr virus IgG seropositivity
Exposure	GCST90006898	Epstein–Barr virus EA-D antibody levels
Exposure	GCST90006899	Epstein–Barr virus EBNA-1 antibody levels
Exposure	GCST90006900	Epstein–Barr virus VCA p18 antibody levels
Exposure	GCST90006901	Epstein–Barr virus ZEBRA antibody levels
Exposure	GCST90006902	Antihuman herpes virus 6 IgG seropositivity
Exposure	GCST90006903	Antihuman herpes virus 6 E1A IgG seropositivity
Exposure	GCST90006904	Human herpes virus 6 IE1A antibody levels
Exposure	GCST90006905	Antihuman herpes virus 6 IE1B IgG seropositivity
Exposure	GCST90006906	Human herpes virus 6 IE1B antibody levels
Exposure	GCST90006907	Human herpes virus 6 p101k antibody levels
Exposure	GCST90006908	Antihuman herpes virus 7 IgG seropositivity
Exposure	GCST90006884	Anti-BK polyomavirus IgG seropositivity

Summary statistics were obtained from the GWAS catalog (accession IDs: GCST90006884-GCST90006909) based on the UK Biobank cohort (n = 9724). The table lists the specific antibody response, corresponding pathogen, sample size, number of cases (for seropositivity traits), number of instrumental variables (IVs) used in the analysis, and the mean *F*-statistic for the IVs.

GWAS = genome-wide association study, N = sample size, N cases = number of seropositive cases, IV= instrumental variable, UREA = urea, VCA = viral capsid antigen, ZEBRA = BamHI Z EBV replication activator.

**Table 2:** Characteristics of the genome-wide association study (GWAS) datasets for the 5 allergic diseases (outcomes).

	GWAS ID	Trait name	Cases	Controls	Populations
Outcome	finngen_R12_ALLERG_ASTHMA	Allergic asthma	13,450	2,70,290	Europeans
Outcome	finngen_R12_H7_ALLERGICCONJUNCTIVITIS	Allergic conjunctivitis	29,791	4,70,557	Europeans
Outcome	finngen_R12_L12_ATOPIC	Atopic dermatitis	31,245	4,32,874	Europeans
Outcome	finngen_R12_ALLERG_RHINITIS	Allergic rhinitis	15,569	4,74,650	Europeans
Outcome	finngen_R12_L12_URTICA_ALLERG	Allergic urticaria	3315	4,82,901	Europeans

Summary statistics were obtained from the FinnGen consortium (R12 release). The table details the specific disease, its abbreviation, sample size, number of cases, number of controls, and the corresponding FinnGen phenotype code.

AA = allergic asthma, AC = allergic conjunctivitis, AD = atopic dermatitis, AR = allergic rhinitis, AU = allergic urticaria, GWAS = genome-wide association study, N = total sample size.

### 2.3. IV selection

To ensure that the screened SNPs are closely related to the exposure factors, we have established the following screening criteria. Firstly, to ensure the strength of IVs while accounting for the limited number of genome-wide significant SNPs for some exposures, we set a significance threshold of *P* < 5 × 10⁻⁶ to identify a sufficient number of exposure-associated SNPs. Next, we applied a linkage disequilibrium threshold with *R*^2^ < 0.001 and an aggregation distance of 10,000 kb to enhance randomness.^[22]^ In addition, to minimize bias in genotyping techniques, palindromic SNPs were excluded. To further ensure strong correlation, we need an *F*-statistic greater than 10 to exclude weak IVs.^[23]^ The *F*-statistic for each SNP was calculated using the formula *F* = β^2^/SE^2^, where β is the β coefficient and SE is the standard error.

### 2.4. Statistical analysis

#### 2.4.1. Forward MR analysis

Using selected IVs, 2-sample MR analysis was performed for 5 allergic diseases with the TwoSampleMR and Mendelian randomization-the pleiotropic residual sum and outlier test (MR-PRESSO) packages in R (version 4.3.3). MR analysis was performed using 5 methods: inverse-variance weighted (IVW) as the primary method, supplemented by MR-Egger, weighted median and weighted mode. The IVW method, inspired by meta-analysis approaches, combines Wald ratio estimates across IVs through statistical pooling or weighted linear regression. By employing inverse-variance weighting, it reduces variance and favors the most statistically powerful estimates, typically providing the highest statistical power.^[24]^ The MR-Egger method accommodates invalid IVs and delivers more valid causal effect estimates in the presence of pleiotropic effects.^[25]^ The weighted median method provides robust estimates when directional horizontal pleiotropy is present in some IVs (<50%).^[26]^ the weighted mode exhibits sensitivity to heterogeneity.^[27]^ Additionally, we visualized the effects of all forth analytical methods through scatter plots and forest plots, with fitted results intuitively reflecting effect trends.

#### 2.4.2. Reverse MR analysis

We employed reverse MR to investigate the bidirectional causal relationship between allergic diseases and AIR, using identical analytical methods and procedures as described above. Allergic diseases served as the exposure, while AIR were treated as the outcome. This reverse analysis also served to verify and exclude potential reverse causation.

#### 2.4.3. Sensitivity analysis

To assess the robustness and validity of our MR estimates, we performed a comprehensive set of sensitivity analyses: Cochran’s *Q* test: used to quantify heterogeneity among the causal estimates of individual SNPs. A significant *Q* statistic (*P* < .05) indicates the presence of heterogeneity, which may suggest pleiotropy. MR-Egger intercept test: used to directly assess directional horizontal pleiotropy. A nonzero intercept (*P* < .05) suggests that pleiotropy is biasing the IVW estimate. MR-PRESSO global test: used as an additional method to detect horizontal pleiotropy by identifying outliers. Leave-one-out analysis: Iteratively removing each SNP to determine if the overall causal estimate is driven by a single influential variant. Funnel plots: Visually inspected for asymmetry, which can indicate potential pleiotropy.

## 3. Results

### 3.1. Causal effects of AIR on AA

Analyses using robust methods – specifically, the weighted median (OR = 1.118, 95% CI = 1.024–1.221, *P* = .013) and weighted mode (OR = 1.158, 95% CI = 1.021–1.312, *P* = .036) – supported a potential causal relationship between polyomavirus 2 JC VP1 antibody levels and AA, indicating a positive association. MR-Egger analysis revealed that Antihuman herpes virus 7 (HHV-7) IgG seropositivity (OR = 2.273, 95% CI = 1.084–4.766, *P* = .041) was positively correlated with AA. No significant heterogeneity or horizontal pleiotropy was observed in the results (all *P* > .05), as detailed in Tables S1 and S6, Supplemental Digital Content, https://links.lww.com/MD/Q590.

### 3.2. Causal effects of AIR on AC

IVW analysis revealed *Toxoplasma gondii (T gondii*) p22 antibody positivity as a significant protective factor for AC (OR = 0.966, 95% CI = 0.936–0.995, *P* = .011), with consistent stability demonstrated by weighted median (OR = 0.953, 95% CI = 0.913–0.994, *P* = .026) and weighted mode methods (OR = 0.971, 95% CI = 0.949–0.993, *P* = .040). No significant heterogeneity or horizontal pleiotropy was detected (*P* > .05). MR-Egger indicated Cytomegalovirus pp28 antibody levels negatively correlated with AC (OR = 0.809, 95% CI = 0.709–0.924, *P* = .012) without heterogeneity (*P* > .05), though horizontal pleiotropy was observed (*P* < .05; Tables S2 and S6, Supplemental Digital Content, https://links.lww.com/MD/Q590).

### 3.3. Causal effects of AIR on AD

Weighted median (OR = 1.080, 95% CI = 1.024–1.139, *P* = .004) and weighted mode (OR = 1.077, 95% CI = 1.021–1.136, *P* = .017) revealed Epstein–Barr virus (EBV) EBNA-1 antibody levels positively correlated with AD. MR-Egger demonstrated *C trachomatis* momp A antibody levels (OR = 0.952, 95% CI = 0.912–0.995, *P* = .049) negatively correlated with AD. Moreover, all the above results showed no significant heterogeneity or horizontal pleiotropy (all *P* > .05; Tables S3 and S6, Supplemental Digital Content, https://links.lww.com/MD/Q590).

### 3.4. Causal effects of AIR on AR

IVW identified *H pylori* CagA antibody levels (OR = 1.053, 95% CI = 1.008–1.101, *P* = .020) and *H pylori* OMP antibody levels (OR = 1.111, 95% CI = 1.019–1.211, *P* = .017) as risk factors for AR. Weighted median (OR = 0.919, 95% CI = 0.856–0.987, *P* = .020) and weighted mode (OR = 0.904, 95% CI = 0.837–0.976, *P* = .020) showed EBV EBNA-1 antibody levels negatively correlated with AR. And all the above results have passed the heterogeneity and horizontal pleiotropy tests (*P* > .05). MR-Egger indicated *H pylori* VacA antibody levels (OR = 0.894, 95% CI = 0.837–0.994, *P* = .006) negatively correlated with AR, without heterogeneity (*P* > .05) but with pleiotropy (*P* < .05; Tables S4 and S6, Supplemental Digital Content, https://links.lww.com/MD/Q590).

### 3.5. Causal effects of AIR on AU

IVW confirmed *H pylori* Catalase antibody levels (OR = 1.251, 95% CI = 1.062–1.473, *P* = .020) positivity as a risk factor for AU, reinforced by MR-Egger (OR = 1.143, 95% CI = 1.006–1.298, *P* = .031) and weighted median (OR = 1.112, 95% CI = 1.017–1.217, *P* = .040). IVW analysis demonstrated that herpes simplex virus 2 (HSV-2) mgG-1 antibody levels (OR = 1.095, 95% CI = 1.017–1.180, *P* = .016) were positively associated with AU, while Antihuman herpes virus 6 (HHV-6) IE1B IgG seropositivity (OR = 0.562, 95% CI = 0.333–0.947, *P* = .031) showed a negative association. All without heterogeneity/pleiotropy (*P* > .05; Tables S5 and S6, Supplemental Digital Content, https://links.lww.com/MD/Q590).

**Figure 2.**
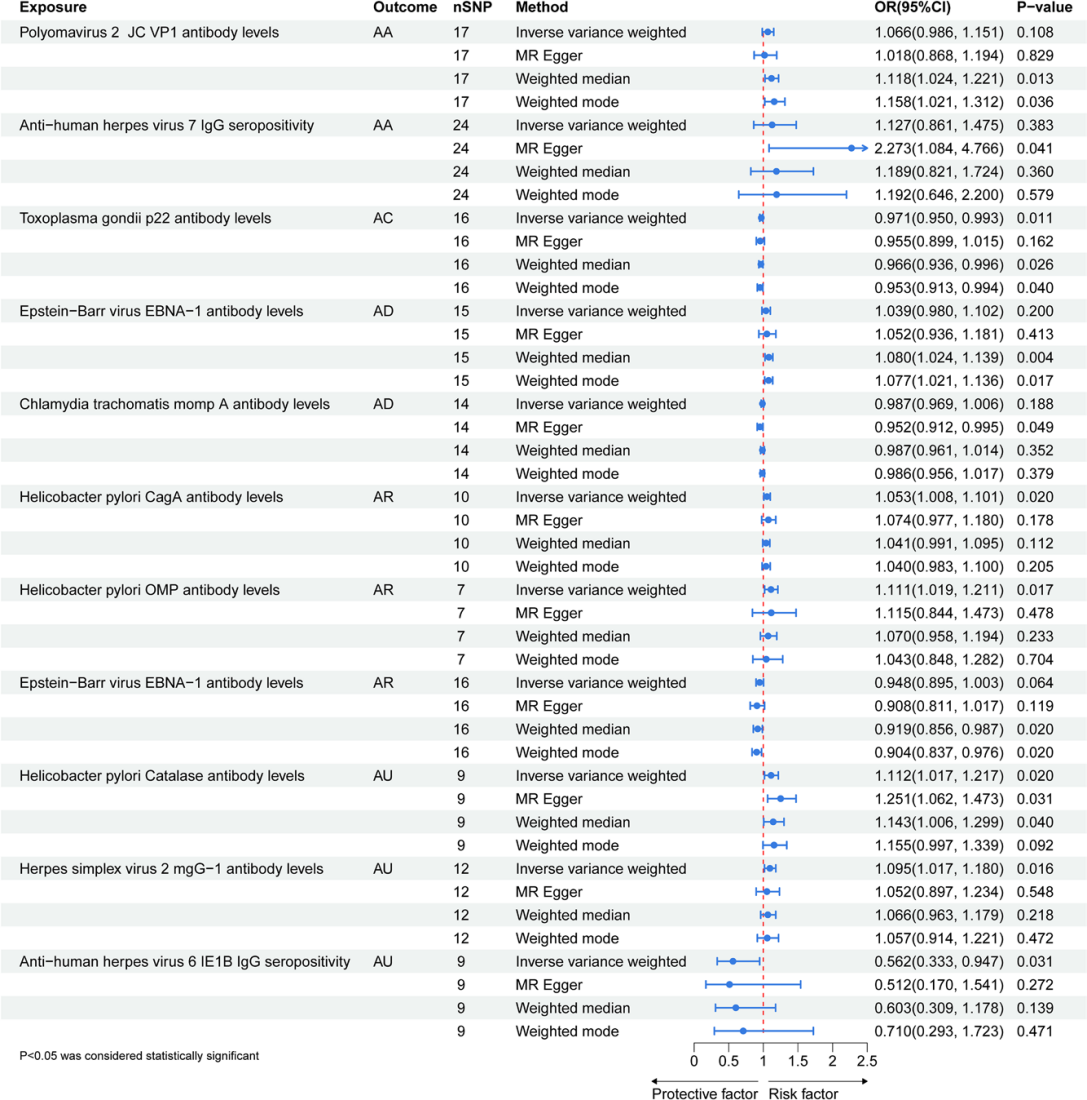
Forest plot of the significant causal associations between antibody-mediated immune responses and 5 allergic diseases identified by the inverse-variance weighted (IVW) method. Note: Each point represents the odds ratio (OR) for a specific exposure-outcome pair, and the horizontal lines represent the 95% confidence intervals (95% CI). The dashed vertical line indicates the null effect (OR = 1). Associations with 95% CIs that do not cross the null line are considered statistically significant. Effect sizes >1 indicate increased risk, while those <1 indicate protection. AA = allergic asthma, AC = allergic conjunctivitis, AD = atopic dermatitis, AR = allergic rhinitis, AU = allergic urticaria, CI = confidence interval, EBNA-1 = Epstein-Barr nuclear antigen 1, IgG = immunoglobulin G, IVW = inverse-variance weighted, JC VP1 = John Cunningham virus viral protein 1, MR = Mendelian randomization, OMP = outer membrane protein, OR = odds ratio, SNP = single-nucleotide polymorphism.

**Figure 3.**
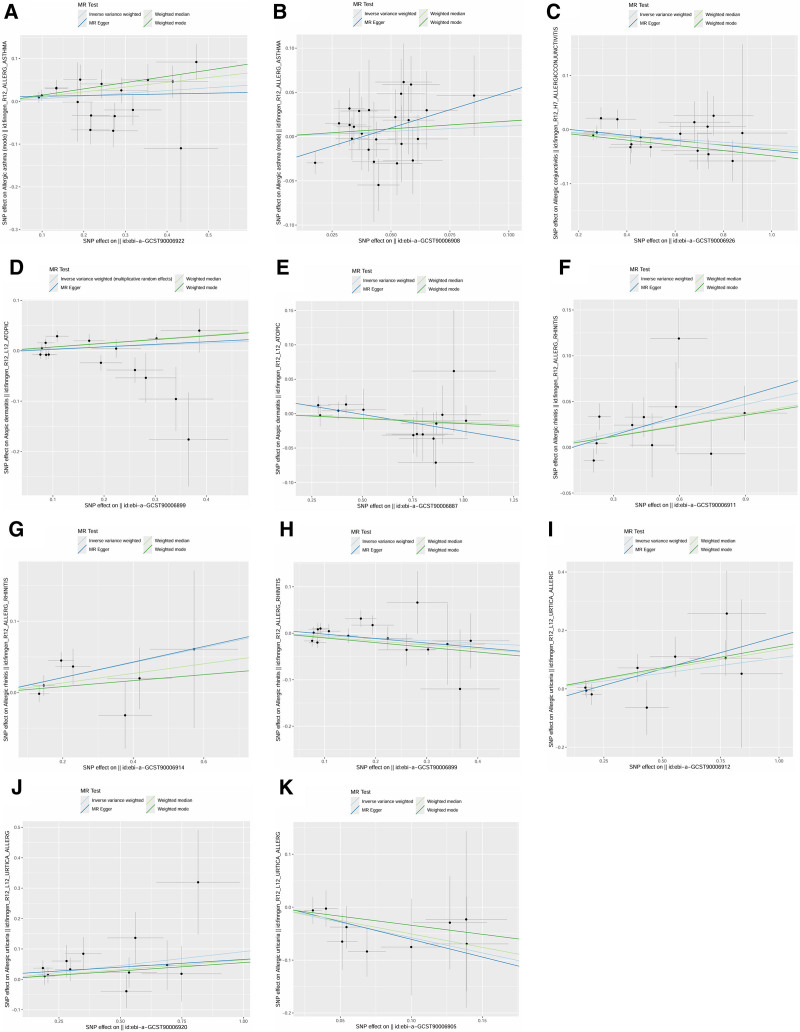
Scatter plots between antibody-mediated immune responses and allergic diseases using univariable MR and bidirectional MR. Note: Each plot shows the SNP-effect estimates on the exposure (*x*-axis) against the SNP-effect estimates on the outcome (*y*-axis). The slopes of the fitted lines represent the causal estimate of each Mendelian randomization (MR) method applied: inverse variance weighted (IVW, light blue line), MR-Egger (dark blue line), weighted median (light green line), weighted mode (dark green line).. The consistency in the direction of the slopes across methods strengthens the evidence for a causal relationship. (A) Polyomavirus 2 JC VP1 antibody levels and AA; (B) antihuman herpes virus 7 IgG seropositivity and AA; (C) *Toxoplasma gondii* p22 antibody levels and AC; (D) Epstein–Barr virus EBNA-1 antibody levels and AD; (E) *Chlamydia trachomatis* momp A antibody levels and AD; (F) *Helicobacter pylori* CagA antibody levels and AR; (G) *Helicobacter pylori* OMP antibody levels and AR; (H) Epstein–Barr virus EBNA-1 antibody levels and AR; (I) *Helicobacter pylori* Catalase antibody levels and AU; (J) herpes simplex virus 2 mgG-1 antibody levels and AU; (K) Antihuman herpes virus 6 IE1B IgG seropositivity and Abbreviation: AA = allergic asthma, AC = allergic conjunctivitis, AD = atopic dermatitis, AR = allergic rhinitis, AU = allergic urticaria, MR = Mendelian randomization, SNP = single-nucleotide polymorphism.

**Figure 4.**
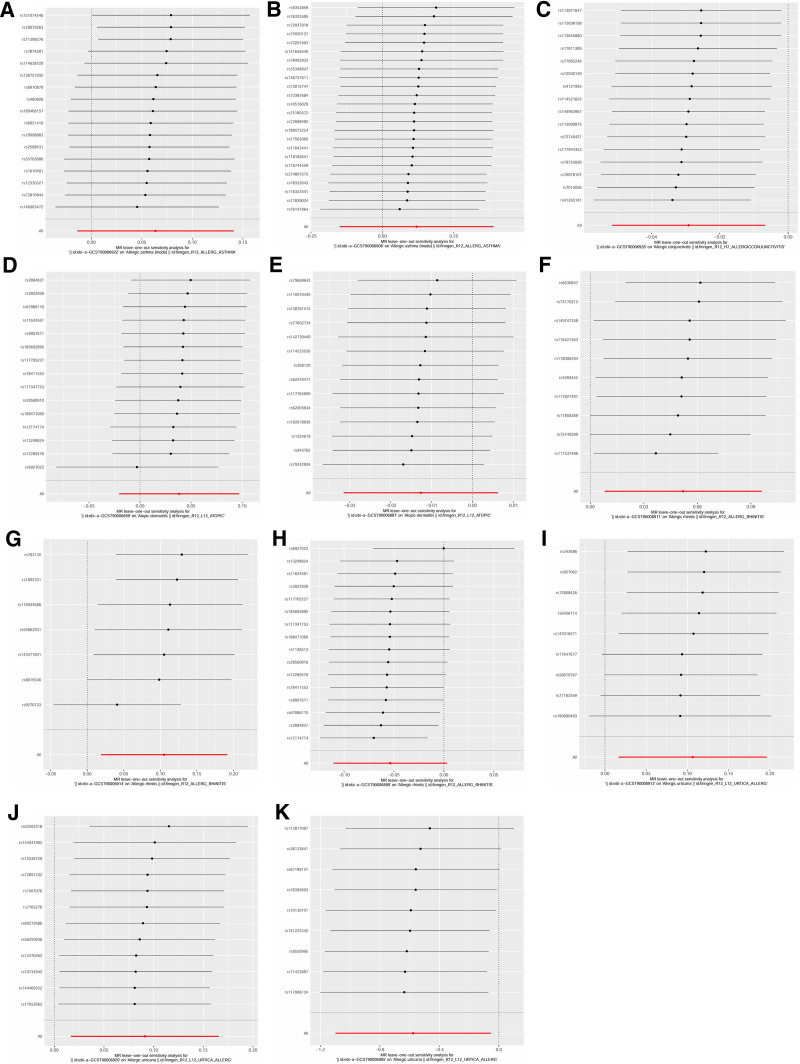
Leave-one-out plots between antibody-mediated immune responses and allergic diseases using univariable MR and bidirectional MR. Note: Each plot shows the causal estimate (red dot) and 95% confidence interval (vertical line) from the inverse-variance weighted (IVW) method when sequentially removing 1 single-nucleotide polymorphism (SNP) at a time. The overall IVW estimate with all SNPs included is represented by a blue horizontal line. The stability of the causal estimate regardless of which SNP is omitted indicates that the results are not driven by any single influential variant. (A) Polyomavirus 2 JC VP1 antibody levels and AA; (B) antihuman herpes virus 7 IgG seropositivity and AA; (C) *Toxoplasma gondii* p22 antibody levels and AC; (D) Epstein–Barr virus EBNA-1 antibody levels and AD; (E) *Chlamydia trachomatis* momp A antibody levels and AD; (F) *Helicobacter pylori* CagA antibody levels and AR; (G) *Helicobacter pylori* OMP antibody levels and AR; (H) Epstein–Barr virus EBNA-1 antibody levels and AR; (I) *Helicobacter pylori* Catalase antibody levels and AU; (J) herpes simplex virus 2 mgG-1 antibody levels and AU; (K) Antihuman herpes virus 6 IE1B IgG seropositivity and AU. AA = allergic asthma, AC = allergic conjunctivitis, AD = atopic dermatitis, AR = allergic rhinitis, AU = allergic urticaria, IVW = inverse-variance weighted, MR = Mendelian randomization, SNP = single-nucleotide polymorphism.

**Figure 5.**
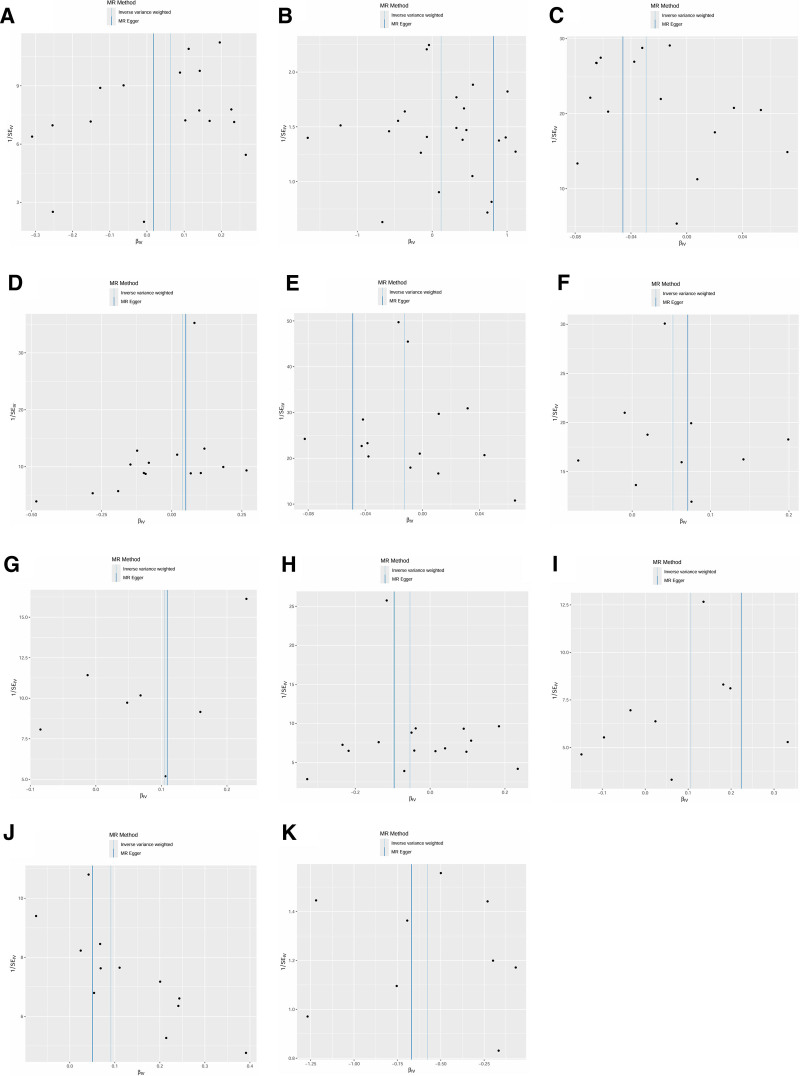
Funnel plots between antibody-mediated immune responses and allergic diseases using univariable MR and bidirectional MR. Note: The funnel plot displays the precision (1/SE) of each SNP’s causal estimate against the estimate itself. In the absence of significant directional pleiotropy, the plot should be symmetrical around the overall IVW causal estimate (vertical dashed line). Asymmetry suggests that the causal estimate might be biased by pleiotropic effects. (A) Polyomavirus 2 JC VP1 antibody levels and AA; (B) antihuman herpes virus 7 IgG seropositivity and AA; (C) *Toxoplasma gondii* p22 antibody levels and AC; (D) Epstein–Barr virus EBNA-1 antibody levels and AD; (E) *Chlamydia trachomatis* momp A antibody levels and AD; (F) *Helicobacter pylori* CagA antibody levels and AR; (G) *Helicobacter pylori* OMP antibody levels and AR; (H) Epstein–Barr virus EBNA-1 antibody levels and AR; (I) *Helicobacter pylori* Catalase antibody levels and AU; (J) herpes simplex virus 2 mgG-1 antibody levels and AU; (K) antihuman herpes virus 6 IE1B IgG seropositivity and AU. AA = allergic asthma, AC = allergic conjunctivitis, AD = atopic dermatitis, AR = allergic rhinitis, AU = allergic urticaria, IVW = inverse-variance weighted, MR= Mendelian randomization, SE = standard error, SNP = single-nucleotide polymorphism.

Significant causal relationships between AIR and the 5 allergic diseases are clearly visualized in Figure 2. The reported associations and corresponding heterogeneity/horizontal pleiotropy tests are detailed in Table S6, Supplemental Digital Content, https://links.lww.com/MD/Q590. Additionally, scatter plots (Fig. 3) demonstrate the causal relationship between individual SNPs associated with AIR and each allergic disease, highlighting risk-increasing variants. Leave-one-out sensitivity analysis (Fig. 4) confirmed that no single SNP substantially altered the overall causal effects of AIR on allergic diseases, supporting result reliability. Funnel plot symmetry (Fig. 5) indicated no directional bias, further enhancing confidence in these findings.

### 3.6. The causal relationship between 5 allergic diseases and AIR (reverse causal relationship)

Reverse MR analysis using IVW revealed that genetically predicted AA was positively associated with anti-*H pylori* IgG seropositivity (OR = 1.034, 95% CI = 1.004–1.065, *P* = .026), indicating that asthma onset influenced by genetic variants enhances this AIR. No significant heterogeneity or horizontal pleiotropy was detected (*P* > .05). Conversely, no associations were observed between AC, AD, AR, AU and any AIR in reverse MR analyses. Detailed results are provided in Table S7, Supplemental Digital Content, https://links.lww.com/MD/Q590.

## 4. Discussion

To our knowledge, this is the first comprehensive bidirectional MR investigation to systematically assess the causal relationships between a wide array of AIR and 5 major allergic diseases. Second, the methodological rigor enhances the reliability of our findings. Furthermore, we employed strict criteria (*F*-statistic > 10) to minimize weak instrument bias. Lastly, the use of large-scale, publicly available GWAS datasets provided substantial statistical power to detect associations. First, we identified polyomavirus 2 JC VP1 antibody levels and Antihuman herpes virus 7 IgG seropositivity as potential risk factors for AA. Polyomavirus 2 (JC virus), a widely disseminated virus, typically reactivates during immunosuppression.^[28]^ Karachaliou et al evaluated associations between DNA viruses and atopic diseases, demonstrating that higher respiratory polyomaviral burden correlates with increased asthma prevalence.^[11]^ AA is characterized by Th2-skewed immunity.^[29]^ Respiratory viruses enhance Th2 response by disrupting the epithelial barrier, activating innate immunity,^[30]^ and polyomavirus may participate in similar pathways. HHV-7 is a T-lymphotropic β-herpesvirus that was isolated from peripheral blood mononuclear cells.^[31]^ The association between HHV-7 IgG seropositivity and AA remains poorly established. Any potential link is likely indirect, possibly mediated through immune dysregulation or virus reactivation mechanisms.^[32]^ Although these associations were not significant under IVW analysis, they remained robust after accounting for pleiotropy and heterogeneity (*P* > .05). Nevertheless, these findings warrant cautious interpretation and further validation through animal models or clinical studies.

This study suggests that robust immune responses to *T gondii* infection may reduce AC risk. *T gondii*, an obligate intracellular protozoan parasite, expresses multiple highly immunogenic surface proteins – p22 being one of 5 major surface antigens during its tachyzoite stage.^[33]^ Notably, Korb et al demonstrated that mucosal application of *T gondii*-derived molecules significantly reduced allergic lung inflammation in mice.^[34]^ Furthermore, certain parasitic infections, such as with Toxocara canis, might even suppress IgE responses to unrelated allergens, highlighting a complex interplay between pathogen exposure and allergic sensitization.^[35]^ Our genetic evidence suggests that Toxoplasma antibody response has a protective effect, suggesting that immune response to these pathogens (possibly formed by lifelong exposure) may be key mediators of this protective pathway. And further confirmed the core principle of the “hygiene hypothesis”: microbial exposure can regulate the immune system to reduce the risk of allergies.^[36]^

Our findings provide genetic evidence supporting a positive association between EBV EBNA-1 antibody levels and AD, alongside a negative correlation with *C trachomatis* momp A antibody levels. EBV is a widely present human herpesvirus, and the EBNA-1 protein expressed during its latent infection phase can reduce its own degradation by inhibiting proteasome function, thereby escaping CD8+ T cell clearance, leading to persistent antigen exposure and chronic inflammation.^[37]^ Such chronic antigen stimulation promotes B cell activation, increases total and allergen-specific IgE production, and could potentially contribute to the exacerbation of AD pathogenesis.^[38]^ Conversely, MOMP is the main outer membrane protein of *C trachomatis*, containing multiple T and B cell epitopes, which can induce the immune system to produce anti-inflammatory cytokines such as IL-10 and TGF-β.^[39]^ These anti-inflammatory cytokines likely inhibit excessive inflammatory responses, protect tissues from damage, and thus reduce the risk of AD. While these mechanisms potentially facilitate long-term pathogen persistence in hosts, their direct impact on allergic disease development remains speculative and warrants cautious interpretation.

Previous studies suggest that early-life microbial exposures, including *H pylori*, may promote normal immune development and regulation, reducing later allergic disease risk.^[40]^ Recent genetic evidence from bidirectional MR analysis also supports a potential protective effect of *H pylori* seropositivity against AR.^[41]^ Paradoxically, our genetic evidence indicates an opposite directionality, implying that sustained humoral immunity targeting specific *H pylori* antigens may constitute a risk factor for AR onset or progression. This divergence crucially stems from differences in exposure definition: whereas the traditional “hygiene hypothesis” and observational studies focus on “*H pylori* infection status” (seropositivity/seronegativity), we examined antibody levels against specific virulence factors (e.g., CagA, OMP). Elevated antibodies typically reflect prior/ongoing infection with virulent strains and intense host-pathogen interactions inducing gastric mucosal inflammation.^[42]^ Thus, our findings do not refute the overall protective role of microbial exposure but reveal that specific *H pylori* exposures – particularly virulent strains or phases eliciting persistent immune activation – may exert pro-allergic effects. In addition, EBV EBNA-1 antibody may be involved in the regulation of Th2 lymphocytes and anti-inflammatory cytokines, and reduce susceptibility to AR.^[43]^ These all emphasize their importance as immune response biomarkers.

Our MR analysis identified *H pylori* Catalase antibody seropositivity as a significant risk factor for AU. Catalase, a key virulence enzyme enabling *H pylori* to resist host oxidative stress, reflects persistent infection and gastric mucosal inflammation when antibodies are elevated. Such infection induces systemic pro-inflammatory cytokines (e.g., IL-6, TNF-α), triggering mast cell degranulation that directly increases histamine and tryptase levels – core mediators of urticarial pathogenesis.^[44]^ Beyond its role in digestion, *H pylori* infection has been increasingly linked to the modulation of systemic autoimmunity, potentially influencing a wide spectrum of immune-mediated conditions.^[45]^ Innovatively, we discovered a positive association between HSV-2 mgG-1 antibody levels and AU risk, potentially involving HSV-2-triggered viral-specific IgE production,^[46]^ a central driver of immediate hypersensitivity reactions.^[47]^ Additionally, we explored HHV-6 IE1B IgG seropositivity in relation to AU. While direct evidence remains limited, established correlations between HHV-6 infection and drug-induced hypersensitivity syndromes warrant further investigation of this relationship.^[48]^

Finally, the reverse MR analysis exclusively indicated that genetically predicted AA enhances anti-*H pylori* IgG seropositivity, with no associations observed for other allergic diseases and AIR. This rules out reverse causation for non-asthma outcomes and further validates the forward MR results (exposure→outcome), thereby preventing causal direction misattribution. Collectively, our MR findings suggest that the role of pathogen-specific immune responses in allergic diseases is not monolithic. It may be governed by a balance between 2 opposing forces: Pro-allergic effects: persistent antibody responses to certain viruses and virulence factors (e.g., from EBV, HSV-2, virulent *H pylori*) may signify chronic immune activation and inflammation that destabilizes immune tolerance and directly amplifies allergic pathways. Protective effects: conversely, robust responses to other pathogens (e.g., *T gondii*) may act as a proxy for prior infection-induced immunomodulation, potentially through the expansion of regulatory cell populations (Tregs, Bregs) that confer long-term protection against aberrant Th2 responses. This dichotomy underscores the importance of moving beyond mere “seropositivity” to a more nuanced understanding of which specific antigens are targeted and what quality of immune response they elicit.

Several limitations warrant consideration. Our study must be interpreted in the context of several methodological limitations inherent to the MR design. First, while we employed rigorous criteria (*F*-statistic > 10) to minimize weak instrument bias, residual bias from undetected weak instruments cannot be entirely ruled out. Second, despite using robust sensitivity analyses (MR-Egger, MR-PRESSO) that indicated no significant horizontal pleiotropy, we cannot definitively exclude the possibility that some genetic variants influence allergic diseases through pathways independent of antibody levels (vertical pleiotropy). Third, as all genetic data were derived from European-ancestry populations, the generalizability of our findings to other ethnic groups is limited and awaits future validation in diverse cohorts. Lastly, the study’s static cross-sectional design based on single-timepoint antibody levels and genotypes data, lacking dynamic observation of the impact of pathogen infection and immune response on disease progression over time. Further validation and improvement are needed in future research.

## 5. Conclusion

This comprehensive analysis provides genetic evidence supporting a potential causal link between AIR and 5 allergic diseases. Our research emphasizes the important role of respiratory viruses and *H pylori* in these allergic diseases, indicating that genetically predicted antibody levels may be related to altered disease risk. Understanding these pathogen-immune interactions may provide new insights for developing treatment strategies, emphasizing the importance of considering microbial infections in the context of allergic diseases. Future research should focus on elucidating the specific immune pathways involved in these associations and exploring potential therapeutic interventions targeting these immune responses.

## Acknowledgments

The author heartfeltly thanks the researchers who conduct GWAS studies and openly shared their summary statistics.

## Author contributions

**Conceptualization:** Lina Leng.

**Data curation:** Lina Leng.

**Formal analysis:** Xiaoli Li.

**Funding acquisition:** Quanyi Tang.

**Investigation:** Lina Leng, Tao Xu, Quanyi Tang.

**Methodology:** Ying Li.

**Project administration:** Lina Leng.

**Resources:** Lina Leng.

**Supervision:** Tao Xu.

**Validation:** Tao Xu.

**Visualization:** Quanyi Tang, Ying Li.

**Writing – original draft:** Lina Leng.

**Writing – review & editing:** Xiaoli Li.

## Supplementary Material



## References

[1] Huang PY Huang YH Guo MM Chang LS Kuo HC. Kawasaki disease and allergic diseases. Front Pediatr. 2020;8:614386. 33490002 10.3389/fped.2020.614386 PMC7817814

[2] Liao Y Zhang QY Li HX Wang YL Liu P Du JB. Co-morbidity of vasovagal syncope and postural tachycardia syndrome with allergic diseases in children. Beijing Da Xue Xue Bao. 2017;49:783–8. 29045956

[3] Dong M Wang WQ Chen J. Acupuncture regulates the balance of CD4+ T cell subtypes in experimental asthma mice. Chin J Integr Med. 2019;25:617–24. 30519873 10.1007/s11655-018-3055-6

[4] Lv JJ Kong XM Zhao Y. Global, regional and national epidemiology of allergic disorders in children from 1990 to 2019: findings from the Global Burden of Disease study 2019. BMJ Open. 2024;14:e080612. 10.1136/bmjopen-2023-080612 PMC11015187 38589255

[5] GBD 2019 Diseases and Injuries Collaborators. Global burden of 369 diseases and injuries in 204 countries and territories, 1990-2019: a systematic analysis for the Global Burden of Disease study 2019. Lancet. 2020; 396:1204–22. 33069326 10.1016/S0140-6736(20)30925-9 PMC7567026

[6] Spergel JM Du Toit G Davis CM. Might biologics serve to interrupt the atopic march? J Allergy Clin Immunol. 2023;151:590–4. 36681581 10.1016/j.jaci.2023.01.001

[7] Seastedt H Nadeau K. Factors by which global warming worsens allergic disease. Ann Allergy Asthma Immunol. 2023;131:694–702. 37689112 10.1016/j.anai.2023.08.610 PMC10873081

[8] Singh AB Kumar P. Climate change and allergic diseases: an overview. Front Allergy. 2022;3:964987. 36310569 10.3389/falgy.2022.964987 PMC9606573

[9] Augustine T Kumar M Al Khodor S van Panhuys N. Microbial dysbiosis tunes the immune response towards allergic disease outcomes. Clin Rev Allergy Immunol. 2023;65:43–71. 35648372 10.1007/s12016-022-08939-9 PMC10326151

[10] Wang J Zhou Y Zhang H. Pathogenesis of allergic diseases and implications for therapeutic interventions. Signal Transduct Target Ther. 2023;8:138. 36964157 10.1038/s41392-023-01344-4 PMC10039055

[11] Karachaliou M de Sanjose S Roumeliotaki T. Heterogeneous associations of polyomaviruses and herpesviruses with allergy-related phenotypes in childhood. Ann Allergy Asthma Immunol. 2021;127:191–9.e3. 33895421 10.1016/j.anai.2021.04.019 PMC8801162

[12] Yu H Robertson ES. Epstein-Barr virus history and pathogenesis. Viruses. 2023;15:714. 36992423 10.3390/v15030714 PMC10056551

[13] Zuo ZT Ma Y Sun Y Bai CQ Ling CH Yuan FL. The protective effects of Helicobacter pylori infection on allergic asthma. Int Arch Allergy Immunol. 2021;182:53–64. 33080611 10.1159/000508330 PMC8117391

[14] Xue Q Li X Li Y Xu J Wu Z Wang J. Dialogue between gastrointestinal tract and skin: new insights into the Helicobacter pylori and atopic dermatitis. Helicobacter. 2021;26:e12771. 33368906 10.1111/hel.12771

[15] Dore MP Meloni G Bassu I Pes GM. Helicobacter pylori infection does not protect against allergic diseases: evidence from a pediatric cohort from Northern Sardinia, Italy. Helicobacter. 2024;29:e13107. 38943311 10.1111/hel.13107

[16] Butler-Laporte G Kreuzer D Nakanishi T Harroud A Forgetta V Richards JB. Genetic determinants of antibody-mediated immune responses to infectious diseases agents: a genome-wide and HLA association study. Open Forum Infect Dis. 2020;7:ofaa450. 33204752 10.1093/ofid/ofaa450 PMC7641500

[17] Visscher PM Wray NR Zhang Q. 10 years of GWAS discovery: biology, function, and translation. Am J Hum Genet. 2017;101:5–22. 28686856 10.1016/j.ajhg.2017.06.005 PMC5501872

[18] Boef AG Dekkers OM le Cessie S. Mendelian randomization studies: a review of the approaches used and the quality of reporting. Int J Epidemiol. 2015;44:496–511. 25953784 10.1093/ije/dyv071

[19] Bowden J Holmes MV. Meta-analysis and Mendelian randomization: a review. Res Synth Methods. 2019;10:486–96. 30861319 10.1002/jrsm.1346 PMC6973275

[20] Hartwig FP Davies NM Hemani G Davey Smith G. Two‐sample Mendelian randomization: avoiding the downsides of a powerful, widely applicable but potentially fallible technique. Int J Epidemiol. 2016;45:1717–26. 28338968 10.1093/ije/dyx028 PMC5722032

[21] Luo F Zhou P Ran X Gu M Zhou S. No evident causal association between Helicobacter pylori infection and colorectal cancer: a bidirectional Mendelian randomization study. Sci Rep. 2023;13:18544. 37899462 10.1038/s41598-023-45545-x PMC10613620

[22] Bowden J Del Greco M F Minelli C Davey Smith G Sheehan NA Thompson JR. Assessing the suitability of summary data for two‐sample Mendelian randomization analyses using MR‐Egger regression: the role of the I2 statistic. Int J Epidemiol. 2016;45:1961–74. 27616674 10.1093/ije/dyw220 PMC5446088

[23] Patel A DiTraglia FJ Zuber V Burgess S. Selecting invalid instruments to improve Mendelian randomization with two-sample summary data. Ann Appl Stat. 2024;18:23–aoas1856. 38737575 10.1214/23-AOAS1856 PMC7615940

[24] Lee YH. Causal association between smoking behavior and the decreased risk of osteoarthritis: a Mendelian randomization. Z Rheumatol. 2019;78:461–6. 29974223 10.1007/s00393-018-0505-7

[25] Burgess S Thompson SG. Interpreting findings from Mendelian randomization using the MR-Egger method. Eur J Epidemiol. 2017;32:377–89. 28527048 10.1007/s10654-017-0255-x PMC5506233

[26] Bowden J Davey Smith G Haycock PC Burgess S. Consistent estimation in Mendelian randomization with some invalid instruments using a weighted median estimator. Genet Epidemiol. 2016;40:304–14. 27061298 10.1002/gepi.21965 PMC4849733

[27] Hartwig FP Davey Smith G Bowden J. Robust inference in summary data Mendelian randomization via the zero modal pleiotropy assumption. Int J Epidemiol. 2017;46:1985–98. 29040600 10.1093/ije/dyx102 PMC5837715

[28] Resiliac J Grayson MH. Atopy and viral infections: could polyomaviruses be to blame? Ann Allergy Asthma Immunol. 2021;127:157–8. 34348849 10.1016/j.anai.2021.05.026

[29] Papadopoulos NG Miligkos M Xepapadaki P. A current perspective of allergic asthma: from mechanisms to management. Handb Exp Pharmacol. 2022;268:69–93. 34085124 10.1007/164_2021_483

[30] Zhang J Zou Y Chen L. Regulatory T cells, a viable target against airway allergic inflammatory responses in asthma. Front Immunol. 2022;13:902318. 35757774 10.3389/fimmu.2022.902318 PMC9226301

[31] Hasan AS Abdulwahab SA Lames K. Prevalence of anti-human herpes virus type 7 IgG positivity rate among children with fever and skin rash in Diyala Province, Iraq. Arch Razi Inst. 2023;78:79–86. 37312736 10.22092/ARI.2022.359149.2381 PMC10258276

[32] Verduyn M Botto G Jaubert J Lier C Flament T Guilleminault L. Serum IgG concentrations in adult patients experiencing virus-induced severe asthma exacerbations. J Allergy Clin Immunol Pract. 2019;7:1507–13.e1. 30654200 10.1016/j.jaip.2018.12.028 PMC7104119

[33] Drinić M Wagner A Sarate P. Toxoplasma gondii tachyzoite-extract acts as a potent immunomodulator against allergic sensitization and airway inflammation. Sci Rep. 2017;7:15211. 29123241 10.1038/s41598-017-15663-4 PMC5680314

[34] Korb E Drinić M Wagner A. Reduction of allergic lung disease by mucosal application of Toxoplasma gondii-derived molecules: possible role of carbohydrates. Front Immunol. 2021;11:612766. 33776987 10.3389/fimmu.2020.612766 PMC7988086

[35] Hodžić A Mateos-Hernández L Fréalle E. Infection with Toxocara canis inhibits the production of IgE antibodies to α-Gal in humans: towards a conceptual framework of the hygiene hypothesis? Vaccines (Basel). 2020;8:167. 32268573 10.3390/vaccines8020167 PMC7349341

[36] Kim S Ndwandwe C Devotta H Kareem L Yao L O’Mahony L. Role of the microbiome in regulation of the immune system. Allergol Int. 2025;74:187–96. 39955207 10.1016/j.alit.2024.12.006

[37] Fuxench ZCC Mitra N Hoffstad OJ Phillips EJ Margolis DJ. Association between atopic dermatitis, autoimmune illnesses, Epstein-Barr virus, and cytomegalovirus. Arch Dermatol Res. 2023;315:2689–92. 37233764 10.1007/s00403-023-02648-9

[38] Yamada M Ishikawa Y Imadome KI. Hypersensitivity to mosquito bites: a versatile Epstein-Barr virus disease with allergy, inflammation, and malignancy. Allergol Int. 2021;70:430–8. 34334322 10.1016/j.alit.2021.07.002

[39] Shetty S Dash S Kumar A Vishwanath S Kini SG Brand A. Immunoinformatics design of a multi-epitope vaccine for Chlamydia trachomatis major outer membrane proteins. Sci Rep. 2024;14:29919. 39623035 10.1038/s41598-024-81736-w PMC11612408

[40] Akiner U Yener HM Gozen ED Kuzu SB Canakcioglu S. Helicobacter pylori in allergic and non-allergic rhinitis does play a protective or causative role? Eur Arch Otorhinolaryngol. 2020;277:141–5. 31555919 10.1007/s00405-019-05659-3

[41] Cao W Chen X Li X Wang Y. Use of bidirectional Mendelian randomization to unveil the association between antibody-mediated immune responses to infectious agents and allergic rhinitis. Hum Vaccin Immunother. 2025;21:2523090. 40625166 10.1080/21645515.2025.2523090 PMC12239776

[42] Sain S Solanki B Kumar N. *Helicobacter pylori* CagA and CagT antibodies arrest the translocation of CagA into gastric epithelial cells. 3 Biotech. 2025;15:179. 10.1007/s13205-025-04343-0 PMC12092867 40406400

[43] Zubchenko S Kril I Potemkina H. Low level of advanced glycation end products in serum of patients with allergic rhinitis and chronic Epstein-Barr virus infection at different stages of virus persistence. J Immunol Res. 2022;2022:4363927. 36405008 10.1155/2022/4363927 PMC9674411

[44] Williams PV. Pharmacologic management of chronic urticaria in pediatric patients: the gap between guidelines and practice. Paediatr Drugs. 2020;22:21–8. 31858489 10.1007/s40272-019-00365-3

[45] Wang L Cao ZM Zhang LL. *Helicobacter pylori* and autoimmune diseases: involving multiple systems. Front Immunol. 2022;13:833424. 35222423 10.3389/fimmu.2022.833424 PMC8866759

[46] Portilho AI Silva VO Brigido LFM De Gaspari E. Should we advance our understanding of immunoglobulin E in viral immunity? Immunology. 2025;176:312–21. 40515421 10.1111/imm.70007 PMC12500387

[47] López-Fandiño R. Role of human antigen-specific T cells in tolerance and IgE-mediated allergic reactions to food. Front Immunol. 2025;16:1595696. 40574856 10.3389/fimmu.2025.1595696 PMC12197947

[48] Mitsui Y Shinkuma S Nakamura-Nishimura Y. Serum soluble OX40 as a diagnostic and prognostic biomarker for drug-induced hypersensitivity syndrome/drug reaction with eosinophilia and systemic symptoms. J Allergy Clin Immunol Pract. 2022;10:558–65.e4. 34757063 10.1016/j.jaip.2021.10.042

